# How to make students satisfied with digital teaching? Investigative results from teaching evaluations in Gynecology and Obstetrics

**DOI:** 10.1007/s00404-022-06645-7

**Published:** 2022-07-19

**Authors:** Steffen Tietz, Laura Bodenbeck, Fabian Riedel, Markus Wallwiener, André Hennigs, Sabine Heublein

**Affiliations:** 1 Department of Gynecology and Obstetrics, Heidelberg University Hospital, Im Neuenheimer Feld 440, 69120 Heidelberg, Germany; 2 Department of Psychology, Heidelberg University, Heidelberg, Germany

**Keywords:** COVID-19, Teaching evaluation, Satisfaction with digital teaching, Gynecology and obstetrics

## Abstract

**Purpose:**

The aim of this study was to investigate whether students’ attitude towards online learning in Gynecology and Obstetrics changed during the COVID-19 pandemic. We further examined which variables impacted students’ satisfaction with digital learning.

**Methods:**

A specifically developed questionnaire was used from June 2020–July 2021 for *N* = 234 medical students participating in the course “Gynecology and Obstetrics” at University of Heidelberg. Thirty-five items were repeatedly applied in different cohorts to assess structure- and content-related quality of teaching. In addition, their influence on overall satisfaction with digital teaching was analyzed by applying investigative analyses like multiple regression and extreme group comparisons.

**Results:**

Especially items associated with content-related quality of teaching (*β* = 0.24), organization of teaching (*β* = 0.25) and subjective learning success (*β* = 0.27) seemed to be relevant predictors for overall satisfaction with courses. Fears and changes due to the pandemic situation also played a role for a subgroup of students. Aspects linked to technical quality of teaching, interactions with teachers and students or advantages of web-based learning appeared to play a subordinate role for overall satisfaction with digital teaching. Comparisons of ratings over time revealed that teaching evaluations almost remained the same.

**Conclusion:**

Our results give several hints regarding how digital teaching should be designed and how it can be improved. Further studies are needed to validate our results and to develop methods to improve digital teaching in medicine.

**Supplementary Information:**

The online version contains supplementary material available at 10.1007/s00404-022-06645-7.

## Introduction

Due to the COVID-19 pandemic universities around the globe were forced to fundamentally change their teaching and learning environment [1]. To prevent or at least limit further spread of the virus, synchronous and asynchronous online teaching has been widely implemented since early 2020. Also, in Germany face-to-face courses had to be suspended as much as reasonably applicable beginning in March 2020 and were only recently and just temporarily able to be resumed in the winter semester 2021/2022 [2].

There have been some initiatives to promote digital learning options before the outbreak of COVID-19 [3, 4], but the pandemic situation forced an acceleration in accepting these technologies for university teaching purposes [5, 6].

The sudden switch form face-to-face to online formats turned out to be challenging for both teachers and students [7, 8]. For example, a longitudinal study of Li et al. [9] revealed that negative affect and anxiety scores increased among Chinese college students during the COVID-19 outbreak. Furthermore, a study by Wang and Zhao [10] found higher anxiety scores among college students during COVID-19 than before, suggesting that fears and worries due to COVID-19 could be an important factor that should be considered in teaching.

Nevertheless, several authors report that digital teaching in medicine during COVID-19 would have been assessed quite positively [11, 12]. Positive effects could be also observed on learning success [13] and so it seems reasonable to consider both disadvantages and advantages of digital teaching in evaluations.

Most observations depicted above were made during the onset of the pandemic. Since we are already facing COVID-19 related restrictions for more than two years, students’ attitude towards online learning might have changed over time. However, relatively few studies have addressed this aspect so far, although studies suggest that teaching elements could be important in the future [14], even independent from COVID-19. We thus investigated whether students’ attitude towards online learning has changed over time.

Considering both challenges and chances related to online learning, students’ satisfaction turned out to be a critical indicator of learning success [15, 16]. Hence it seems to be important to increase satisfaction of medicine students with courses. Another positive effect of higher student satisfaction in medicine might be to motivate students for becoming a doctor later and thus to counteract the shortage of doctors in many countries [17], but this hypothesis must be tested in further studies. To build up an effective learning environment and to meet students’ demands, factors that determine satisfaction with online learning need to be defined.

To increase the student to teacher ratio and to reduce cohort sizes, the clinical semesters (up to 200 students) at University of Heidelberg are regularly split up into four sub-cohorts (30–50 students per sub-cohort). Each sub-cohort is trained in Gynecology and Obstetrics for four weeks. Hence, the course is repeated four times per half-year.

Under pre-pandemic conditions, the curriculum for medical students in Gynecology and Obstetrics at Heidelberg University consisted of lectures, seminars, practical trainings, and clinical clerkships (“Blockpraktika”). Lectures took place at a lecture hall in a teacher-centered style and usually involved voluntary attendance, while compulsory small-group seminars (approx. 20–30 students) were designed for interactive discussions. Practical courses and clerkships provided bedside and hands-on clinical teaching. A written examination and an Objective Structured Clinical Examination (OSCE) concluded a course.

During the restrictions in the pandemic, face-to-face courses and practical trainings had to be suspended totally in Germany (first wave of pandemic) or partly (later waves of pandemic). During that time, most lessons were either held via the University’s video platform or were available for students as videos. Videos could be streamed ‘on demand’ within the online teaching platform. The latter also comprised videos of technical procedures (e.g. examinations), surgeries or doctor-patient-talks. Virtual clinical case scenarios and knowledge-self-tests were also available from the online teaching platform. Teachers were available via email or phone to answer students’ questions regarding the online teaching material.

The aim of our study was to evaluate online teaching in the field of Gynecology and Obstetrics during COVID-19 and to identify determinants of students’ satisfaction with courses. Descriptive findings were validated with inference statistics, and analytical questions were addressed, such as whether there are differences across the three first online semesters during COVID-19. Overall, the study has a rather investigative and explorative character.

## Methods

### Data collection

The questionnaire specially compiled for this study was distributed to medical students participating in the four-week course Gynecology and Obstetrics at Heidelberg University. Different cohorts within June 2020–October 2020 and June–July 2021 were assessed at the end of a course. Questionnaires were filled out directly and collected.

### Materials

The teaching evaluation questionnaire consisted of 35 items. In addition to age, gender, media used in the course, future wishes and workload, various aspects of teaching were evaluated. To assess these aspects, several items were employed, all using Likert scales (ranging from 1 = “do not agree”, 2 = “tend not to agree”, 3 = “partly agree”, 4 = “tend to agree” to 5 = “fully agree”). For measuring “content-related quality of teaching” and “fears and changes due to pandemic situation” five items were used. To determine “technical quality of teaching” and “organization of teaching” three items were defined. Finally, “subjective learning success”, “interactions with teachers and students” were assessed by applying four items. To determine the different aspects of teaching, the sums of the corresponding item scores were formed in each case.

As main outcome variable, overall satisfaction with courses was measured by the item “I found the course overall”. Possible answers were “exceptionally poor”, “somewhat poor”, “neutral”, “somewhat good” and “exceptionally good”.

### Inclusion and exclusion criteria

Medical students enrolled at Heidelberg University were assessed during the course “Gynecology and Obstetrics” which is a compulsory course of the clinical section in the 4th or 5th year of medical studies. For each analysis, all data were used. When individuals did not answer all questions or if their answers were ambiguous, data were excluded, but only for those analyses that concerned these data. For the analyses on age dependency and gender dependency, one person each had to be excluded who was the only one to fall into a respective category (only one person indicated to be between 35 and 50 years old and only one person was diverse). Thus, it was not possible to integrate them meaningfully into the inferential statistical analyses, where more people per category are needed.

### Analyses

Descriptive and statistical tests were derived from the raw data using the program “R Studio” (Version 2021.9.0.351) of the R Core Team [18]. Items with similar content were exploratively summarized into different aspects of teaching. These subscales were intercorrelated to analyze the structure of the questionnaire. Reliabilities were calculated to estimate the technical accuracy of measurement and to assess if different aspects of teaching were measured similar reliable. This is important, because otherwise the relationship of a less reliable measured aspect of teaching with overall satisfaction with teaching could be underestimated compared to a more reliable measured aspect of teaching.^1^ Cronbach’s *α* was used to estimate reliability. To analyze the structure of the questionnaire, intercorrelations of the different aspects of teaching were calculated.

One focus of this study was to inferentially validate descriptive results against chance. For example, the dependencies from sample characteristics like age, gender and semester, in which the course was done by the student, were analyzed using *χ*^2^ tests for independent samples. This test allows to investigate whether differences between descriptive values can be secured against chance or whether they could be purely accidental. If differences appear to be significant, the size of this effect can be estimated by calculating Cramers *V*. An advantage of *χ*^2^ tests is, that it is robust against violation of normal distributions.

To predict overall satisfaction with courses, multiple regression analyses were calculated with single variables and different aspects of teaching as predictors. To get a parsimonious model and to find out, which predictors are especially important, the model with the best Akaike Information Criterion [19] was determined. A reason for this is, that with increasing number of predictors the clarification of variance, but also the risk of an overfitting model expands. Some predictor could clarify bit of the variance in the model, but this could be an accidental finding in the sample that is not relevant for the whole population. The aim of the Akaike Information Criterion [19] is to find a good compromise between as much variance clarification as possible and as much parsimony as possible and was used out of these reasons.

Exploratively, also extreme group comparisons between students with extremely high versus extremely low overall satisfaction were calculated. Wilcoxon rank sum tests were used for this, that are robust against violations of normal distribution. Persons *r* was calculated to estimate the effect sizes between significant differences shown by these tests.

## Results

### Sample characteristics

A total of *N* = 234 students filled out the questionnaire. Of these, 132 (56.41%) were female, 101 (43.16%) were male, and 1 (0.43%) was diverse. 147 (62.82%) were under 25 years old, 86 (36.75%) were between 25 and 35 years old, and 1 (0.43%) was between 35 and 50 years old.

### Descriptive analysis of items assessed

In total, 35 items were assessed. Modal values, means and sample sizes for all items on the different aspects of teaching are depicted in Sup Table 1. To increase clearness of the data, items were categorized into 6 thematic groups (Table 1). Within each thematic group item ratings were summed up and then divided by the total number of items. After recoding one invertedly-coded item, all items were scaled in a way that a high numerical value represents a high expression of the respective characteristic. Also reliabilities of the different aspects of teaching were used, ranging from *α* = 0.640 to α = 0.812. Results are shown in Table 1.

**Table 1:** Means and standard deviations of different aspects of teaching

Aspect of teaching	*M*	*SD*	Cronbach’s *α*
Content-related quality of teaching	66.42%	20.00%	0.812
Technical quality of teaching	69.10%	19.41%	0.641
Organization of teaching	60.01%	23.62%	0.704
Advantages of web-based learning	63.48%	21.03%	0.718
Subjective learning success	66.43%	18.25%	0.670
Interaction with teachers and students	53.79%	21.76%	0.768
Fears and changes due to pandemic situation	42.27%	20.88%	0.640

Means (*M*) and standard deviations (SD) values refer to the maximum measurable expression. Cronbach’s *α* is an estimator for reliability of measurement

The answers to items related to the groups “content-related quality of teaching”, “technical quality of teaching”, “organization of teaching” and “advantages of web-based learning” mostly ranged within medium (Likert: 3) or affirmative (Likert: 4) level. This observation applied to 12 out of 15 items. An item on flexibility (“Advantages of web-based learning gave me more flexibility in my life.”) stood out in this pattern with even more agreement. More than two out of three students (68.70%) fully agreed (Likert: 5) to this statement.

The aspect “subjective learning success” provided a more heterogeneous picture, especially when locking at the frequencies of given answers. For example, 23.48% of the students fully agreed and 39.18% tended to agree to the statement that their learning gain was high. On the opposite, 77.63% of the students fully agreed to the statement that without personal contact with patients they would lack practical application of their newly learned skills.

Items concerning the aspect “fears and changes due to pandemic situation” showed that there were students who had worries and fears about studying under pandemic situation, although this was not the case for most of them. The most likely agreement of this aspect was found with the statement that the daily rhythm would have changed due to the self-study. 39.74% fully agreed and 30.13% tended to agree to the corresponding item. For more serious items, such as whether more nervousness was felt before the exam than before past exams, there was descriptively less agreement. To this item 5.70% fully agreed and 11.40% tended to agree.

Apart from the aspect “fears and changes due to pandemic situation”, the aspect “interaction with teachers and students” was the one with descriptively lowest expression. For example, most students tended not to agree to statement, that there would have been enough possibilities to exchange with students during the course.

Overall satisfaction, measured by the item “I found the course overall” was rated as “exceptionally poor” by 6 (2.67%), “somewhat poor” by 27 (12.00%), “neutral” by 79 (35.11%), “somewhat good” by 90 (40.00%), and “exceptionally good” by 23 students (10.22%).

The workload, measured by the item “Compared to corresponding conventional courses, my workload for online teaching is” was rated by 7 students (3.08%) as “very large”, by 37 (16.30%) as “rather large”, 126 (55.51%) as “normal”, 45 (19.82%) as “rather small” and 12 (5.29%) as “very small”. Consequently, most of the students considered the workload during the online course of “Gynecology and Obstetrics” to be similar to the workload during previous courses in a face-to-face semester.

Students also could indicate several future wishes. 87 students (39.55%) indicated “more lectures held online”, 76 (34.55%) “more seminars held online”, 141 (64.09%) “more self-study materials made available online”, and 51 (23.18%) found that “the ratio of components was balanced”.

### Significant intercorrelations of the different aspects of teaching

For analyzing the structure of the evaluation questionnaire used, intercorrelations of the different aspects of teaching were calculated. The correlation matrix with spearman´s rank correlations and significance codes is shown in Table 2. All aspects, apart from “fears and changes due to pandemic situation” were correlated with each other with medium to high correlations in the range of rho = 0.28 to rho = 0.67. These correlations could be secured against chance (*p* < 0.001 for all these correlations). The aspect "fears and changes due to pandemic situation” stood out in this pattern. Correlations with other aspects of teaching were only of a small to medium size, ranging from rho = − 0.11 to rho = − 0.27, but apart from the correlation with “technical quality of teaching”, they were also statistically significant. Overall, the various aspects of teaching appeared to be closely interrelated.

**Table 2:** Correlation matrix of the different aspects of teaching with spearman’s rank correlations

	Technical quality of teaching	Organization of teaching	Advantages of web-based learning	Subjective learning success	Interaction with teachers and students	Fears and changes due to pandemic situation
Content-related quality of teaching	0.46***	0.67***	0.52***	0.65***	0.47***	− 0.26***
Technical quality of teaching		0.53***	0.28***	0.35***	0.34***	− 0.11
Organization of teaching			0.43***	0.47***	0.50***	− 0.22**
Advantages of web-based learning				0.41***	0.44***	− 0.27***
Subjective learning success					0.50***	− 0.25***
Interaction with teachers and students						− 0.18*

Significance codes: + *p* < 0.10, *p < 0.05, **p < 0.01, ***p < 0.001

〹

### Stability of different aspects of teaching over time

Investigative tests were also carried out to determine the extent to which the aspects of teaching depended on time. For this purpose, *χ*^2^ tests for independent samples were calculated with the individual aspects of teaching as dependent variables and the semester in which data were collected (summer semester 2020, winter semester 2020/2021, summer semester 2021) as independent variables. No significant effects showed up (Table 3).

**Table 3:** *p*-values of *χ*^2^ tests to investigate differences in the aspects of teaching depending on sample characteristics

	Semester	Gender		Group of age	
	*p*	*V*	*p*	*V*	*p*
Content-related quality of teaching	0.363		0.924		0.152
Technical quality of teaching	0.726		0.874		0.223
Organization of teaching	0.420		0.080		0.680
Advantages of web-based learning	0.317		0.201		0.468
Subjective learning success	0.297		0.071	0.39	0.001
Interaction with teachers and students	0.230	0.40	0.013		0.426
Fears and changes due to pandemic situation	0.055		0.439		0.473

*V* = Cramer’s *V* (effect size). Only effect sizes with statistical significance are reported

### Independency of different aspects of teaching from sample characteristics

To investigate whether sample characteristics, namely age or gender influenced teaching evaluations, *χ*^2^ tests for independent samples were calculated with the individual aspects of teaching as dependent and the sample characteristics as independent variables. “Interaction with teachers and students” was significantly higher for women (*M* = 55.11%; SD = 21.75%) than for men (*M* = 52.54%; SD = 21.60%) with *χ*^2^ = 19.35, *p* = 0.013 and an effect size of Cramer’s *V* = 0.39. “Subjective learning success” was significantly higher for students aged between 25 and 35 (*M* = 48.96%; SD = 17.65%) than for students younger than 25 (*M* = 43.66%; SD = 18.24%) with *χ*^2^ = 34.84, *p* = 0.001 and an effect size of Cramer’s *V* = 0.40. All other tests did not show any significances (Table 3). Consequently, despite these two significant differences the evaluations seemed to be largely independent from gender and group of age.

### Prediction of overall satisfaction with course

In terms of learning success, overall satisfaction with the course is of particular interest [15, 16]. Thus, we analyzed to what extent overall satisfaction with the course depended on the various aspects of teaching. For this purpose, a multiple regression was calculated (Table 4). The test of the whole model with *F* (7, 194) = 30.35 was statistically significant (*p* < 0.001) and could clarify about half of variance of overall satisfaction with course (*R*^2^ = 52.27% in the sample, respectively, estimated for the population an adjusted *R*^2^ = 50.55%). Only the aspects “Content-related quality of teaching” with a standardized regression coefficient of *β* = 0.24 (*p* = 0.003), “Organization of teaching” with *β* = 0.24 (*p* = 0.001) and “Subjective learning success” with *β* = 0.23 (*p* = 0.001) were significant single predictors in the overall model. It should be considered that reliabilities of the different aspects of teaching were not the same and out of statistical reasons it could be that aspects with less reliability are underestimated in its influence on overall satisfaction with courses. Nevertheless, differences in reliability were note very huge, so that these results were assumed in following analyses.

**Table 4:** Multiple regression of overall satisfaction with course by different aspects of teaching

Predictor	*β*	*σ*	*p*
Intercept	**3.44**	0.05	< 0.001
Content-related quality of teaching	**0.24**	0.08	0.003
Technical quality of teaching	− 0.02	0.05	0.775
Organization of teaching	**0.24**	0.07	0.001
Advantages of web-based learning	0.05	0.06	0.418
Subjective learning success	**0.23**	0.07	0.001
Interaction with teachers and students	0.05	0.06	0.367
Fears and changes due to pandemic situation	0.00	0.05	0.924

*β* = Estimate for standardized regression coefficient. *σ* = Standard deviation error. Significant coefficients (*β*) are printed bold

It turned out, that a model with the three predictors “content-related quality of teaching”, “organization of teaching” and “subjective learning success” represented the best compromise according to the Akaike Information Criterion (Table 5). The whole model with these three predictors was statistically significant with *F* (3, 211) = 69.06 (*p* < 0.001) and could clarify.

**Table 5:** Multiple regression of overall satisfaction with course by different aspects of teaching using the Akaike Information Criterion

Predictor	*β*	*σ*	*p*
Intercept	**3.42**	0.05	< 0.001
Content-related quality of teaching	**0.24**	0.08	0.002
Organization of teaching	**0.25**	0.06	< 0.001
Subjective learning success	**0.27**	0.06	< 0.001

*β* = Estimate for standardized regression coefficient. *σ* = Standard deviation error. Significant coefficients (*β*) are printed bold

Almost half of the variance (*R*^2^ = 49.54% in the sample, respectively, estimated for the population *R*^2^ = 48.83%). All three single predicators were significant: For the “content-related teaching quality” *β* = 0.24 with *p* = 0.002 was found, for the “organization of teaching” *β* = 0.25 with *p* < 0.001 and for the “subjective learning success” *β* = 0.27 with *p* < 0.001.

In addition, a multiple regression was calculated with the single items instead of the aggregated aspects of teaching. Due to the quantity of items, a more parsimonious model was selected directly for this purpose using backward elimination and the Akaike Information Criterion [19] again. The resulting model (Table 6) was statistically significant with *F* (6, 191) = 41.54 (*p* < 0.001) and could clarify more than half of the variance (*R*^2^ = 56.62% of the sample, respectively, an adjusted *R*^2^ = 55.25% of the population variance). Descriptively, the overall satisfaction could be predicted even better by these six single items than by the three blocks reported above.

**Table 6:** Multiple regression of overall satisfaction with course by different items using the Akaike Information Criterion

Predictor	*β*	*σ*	*p*
Intercept	0.00	0.05	1.000
The lectures of this course are well organized	**0.296**	0.06	< 0.001
Doctrinal content is taught in an understandable way	**0.221**	0.07	0.002
With the materials provided, I felt I was adequately prepared for the exam this semester	**0.203**	0.06	< 0.001
The format of the courses with the elements used is well suited to achieve the learning objectives	**0.143**	0.07	0.057
Advantages of web-based learning led to more efficiency in my learning	**0.010**	0.05	0.053
Compared to corresponding conventional courses, my workload for online teaching is…	**− 0.098**	0.05	0.042

*β* = Estimate for standardized regression coefficient. *σ* = Standard deviation error. Significant and marginal significant coefficients (*β*) are printed bold

### Extreme group comparisons

Finally, students with particularly low and particularly high overall satisfaction with course were compared. Two extreme groups were formed for this purpose. The group with particularly low overall satisfaction consisted of all students who rated the course as “exceptionally poor” or “rather poor” (together *N* = 33). The group with particularly high overall satisfaction consisted of those who rated the module as “exceptionally good” (*N* = 23). Differences were not only examined descriptively, but also inferentially tested against chance. For this purpose, Wilcoxon rank sum tests for independent samples with continuity correction were used to examine the various aspects of teaching. Wilcoxon tests were run because they are robust to the violated normal distributions shown by significant Lilliefors tests.

As shown in Table 7 there were statistically significant differences for all aspects of teaching. Students with high overall satisfaction with course rated all aspects of teaching as better, whereas “fears and changes due to pandemic situation” were less compared to students with low overall satisfaction with course.

**Table 7:** Comparison of different aspects of teaching between students with high versus low overall satisfaction with course with Wilcoxon rank sum tests

Aspects of teaching	Low overall satisfaction with course		High overall satisfaction with course		Wilcoxon-Test		
	*M*	SD	*M*	SD	*z*	*r*	*p*
Content-related quality of teaching	0.4500	0.2179	0.8864	0.1125	**− 5.79**	**0.78**	< 0.001
Technical quality of teaching	0.5657	0.2112	0.7765	0.1530	**− 3.69**	**0.50**	< 0.001
Organization of teaching	0.3864	0.2223	0.8261	0.1463	**− 5.63**	**0.75**	< 0.001
Advantages of web-based learning	0.4848	0.2322	0.8267	0.1636	**− 4.82**	**0.65**	< 0.001
Subjective learning success	0.4583	0.1759	0.8234	0.1515	**− 5.50**	**0.74**	< 0.001
Interaction with teachers and students	0.3569	0.1702	0.7232	0.1785	**− 5.19**	**0.72**	< 0.001
Fears and changes due to pandemic situation	0.4938	0.2039	0.3386	0.2198	**− 2.49**	**0.33**	0.013

Significant effects are in bold

To investigative differences of the extreme groups depending on age, gender, or year of study, *χ*^2^ tests for independent samples were used due to the nominal scale level of the dependent variables. However, no statistical significance was found for age (*χ*^2^ = 0.16; *p* = 0.686), gender (*χ*^2^ = 1.69; *p* = 0.194) and year of study (*χ*^2^ = 5.65; *p* = 0.059).

## Discussion

### Discussion of the descriptive analyses and resulting hypotheses

The descriptive expressions of different aspects of teaching (Supplementary Table 1) provide an overview of how online learning was evaluated. However, these values need to be interpreted with caution, since it is not apparent directly whether differences between descriptive values are accidently or can be secured against chance. Therefore, one methodical focus of our study was the inferential statistical validation of findings.

Bearing in mind the limitations of descriptive data, they are still suitable to develop certain hypotheses. Our data presented here suggest that students are rather satisfied with their online classroom. This is not only supported by analysis of the item assessing overall satisfaction with course, but also by several other items on partial aspects of teaching. However, these absolute results must be seen in the context of and compared to student satisfaction in regular face-to-face modules prior to the pandemic, which is not part of this study. Anyway, our study reveals potential starting points for improvements of digital teaching. For example, the aspect “Interactions with teachers and students” could be improved as well as practice-based learning (shown by the item “Without the personal contact to patients, I lacked the practical application of the newly learned”), as can be seen from the medians, which tend to be in the middle or lower range.

Despite these average tendencies, the heterogeneity of responses for some items suggests that teaching during COVID-19 was experienced quite differently. The large effect sizes for different aspects of teaching between the two extreme groups of people with high versus low satisfaction of course confirm this suggestion. Consequently, it could be important, to watch out for the high differences between students, especially when using online formats.

A special feature of our study was, that we also used specific items that addressed the pandemic situation. For example, one of these items (“Self-study has changed my daily rhythm.”) revealed that many students seemed to have changed their daily rhythm due to online courses. Another item (“Advantages of web-based learning gave me more flexibility in my life.”) showed, that many students experienced online teaching as more flexible. Thus, positive, and negative consequences of this change in daily rhythm would be an interesting approach for further studies.

Though most students did not seem to be notably affected by fears and worries due to COVID-19, as assessed by items of the aspect “fears and changes due to the pandemic situation”, a minority of learners seemed to be affected by such worries and fears, at least in a mild form. This is particularly important because of the extreme comparisons showing that there are significant differences in “fears and changes due to pandemic situation” depending on satisfaction with course. An implication of this is that there´s a relevant group of people with notable fears and low satisfaction with course which should not be forgotten. This small group of students at Heidelberg University that was investigated in our study could even underestimate the situation at other Universities. Looking at studies of Li et al. [9] or Wang and Zhao [10] with Chinese students one might suggest that fears and worries during the pandemic could be large at other Universities with different regulations and different economic backgrounds of students. This has far-reaching implications for students’ mental health and should therefore be addressed in further studies.Surprisingly, many students wish for more online learning opportunities in the future, even independent from COVID-19. This result was also found by Riedel et al. [20] and conveys the impression that it could be worthwhile to make greater use of digital teaching formats even independently of COVID-19. Ideally, the implementation of new digital teaching concepts should be scientifically accompanied and evaluated in future studies like the one of Riedel et al. [20]. Our own study gives hints, on what should be of special attention when improving digital teaching.

### What is important for overall satisfaction with course?

Investigative examination of the various aspects of teaching revealed that they are all appreciably correlated. A plausible explanation for this would be that they all contribute to the measurement of a superordinate factor. According to the regression analyses with overall satisfaction as criterion, it seems to be a plausible explanation that this superordinate factor might be overall satisfaction with course. A caveat to the interpretation of all the regression analyses performed is that the normal distribution assumptions of the different aspects of teaching appeared to be violated, but given the large sample, this should hardly limit the explanatory power of these analyses [21].

As can be seen in the parsimonious model in Table 5, especially aspects of “content-related quality of teaching”, “organization of teaching,” and “subjective learning success” are important for overall satisfaction with course. Hence, we suppose that these aspects should be in a particular focus when aiming to improve satisfaction with digital classes. By considering the regression analysis with the single items (Table 6) thematic groups mentioned above may be dissected and interpreted further. On single item level it seems to be particularly important that lectures are well organized, content is taught in an understandable way, materials provided prepare for the exam, the format and elements are suited well to achieve learning objectives, advantages of web-based learning led to more efficiency in learning and the workload compared to corresponding conventional courses shouldn´t get too high.

On the other hand, aspects like “technical quality of teaching”, “interactions with teachers and students” and “advantages of web-based learning” seem to play a subordinate role for overall satisfaction with digital teaching. Consequently, special attention should be paid to those factors that our study proved to be highly relevant for overall satisfaction. This may become of special importance when designing an online curriculum for medical students or when setting up an evaluation questionnaire.

### High similarity of results over time

Remarkably, no statistically significant effects of the various aspects of teaching depending on the year of study was found. Thus, there is no evidence that students became increasingly demoralized and dissatisfied with teaching over the digital semesters, nor is there any evidence that teaching satisfaction would have been appreciably improved by increased knowledge about how to teach completely web based. Of course, it might be that these effects offset each other. In any case, our results give hints that teaching was assessed quite similarly across the first three semesters during COVID-19. The comparatively long study period from June 2020–July 2021 is a particular strength of our study, which sets it apart from other studies like the one from Olmes et al. [11], that only reports data from the summer semester 2020. Our results implicate that assessment of different aspects of online teaching might be quite stable and that results are also important for the future. Age and gender also did not seem to have large effects on the evaluation of digital teaching under the conditions of COVID-19.

### Limitations

Limitations in our results derived from the monocentric approach at Heidelberg University in one particular course during online semesters. That’s why comparisons with other courses like surgery or cardiology are limited as well as comparisons with face-to-face courses before COVID-19. Further research approaches in the future may use additional qualitative methods (for example, structured interviews). It should also be mentioned that overall satisfaction with course was only measured by one item (“I found the course overall”) and it could be interesting to integrate other outcome variables, for example the learning success measured by the grade in examination. This would allow to not only investigate the “subjective learning success”, but also an objective measurement of learning success.

It should be mentioned that the design of the study and analyses were done exploratively. The evaluation questionnaire was self-made and not pretested before. The Likert scales used with an uneven number of answer options might have influenced results due to Error of central tendency. This means that students might have the tendency to often answer “partly agree” in order not to have to decide for lower or higher expression. This would lead to lower reliabilities. Not only low reliability, but also differences in reliability of measurement between different aspects of teaching (Table 1) could have influenced results, because less reliability can lead to underestimation of true correlations and regression coefficients. Nevertheless, differences in reliability were not huge and reliability was acceptable.

### Summary and added value of our study

The added value of our study lies in the combination of several methodological and substantive advantages. For example, inferential statistical methods were used to statistically validate descriptive parameters, and the evaluations were conducted not only in the summer semester of 2020, but also in the two subsequent semesters to be able to examine developmental trajectories. We also used a quite huge sample size. On the content level, the study is characterized by investigating many different aspects of teaching including specific aspects of the pandemic situation.

## Conclusion

Overall, the study shows which aspects are especially important for medical students’ satisfaction with online learning, namely “content-related quality of teaching”, “organization of teaching,” and “subjective learning success”. There does not seem to be any major differences in the evaluation of teaching across the first three online semesters during the pandemic, and age and gender also do not seem to play a major role either. The study provides some practical implications for improving online teaching. The investigative analyses presented may serve as starting points for generating hypotheses and developing further studies. In any case, investigating digital teaching with all its chances and risks is very important, especially because many students would prefer more digital learning opportunities in the future independent from COVID-19.

## Supplementary Information

Below is the link to the electronic supplementary material.

Supplementary file1 (DOCX 17 KB)

## Data Availability

The dataset is available from the corresponding author on reasonable request.

## Footnotes

1 The authors thank Jan Rummel for this helpful note.
