# Supplementary material for: How to make students satisfied with digital teaching? Investigative results from teaching evaluations in Gynecology and Obstetrics
Source: Arch Gynecol Obstet. 2022 Jul 19;306(5):1587–96. doi: 10.1007/s00404-022-06645-7 (PMC9294823; doi:10.1007/s00404-022-06645-7)
Supplement: Supplementary file 1 — Supplementary file1 (DOCX 17 KB) [file 404_2022_6645_MOESM1_ESM.docx]

**Supplementary data**

***Supplementary data 1***

*Modal value, median and sample size of the items on different aspects of teaching*

| Item | Modal value | Median | *N* |
| --- | --- | --- | --- |
| Content-related quality of teaching | | | |
| Doctrinal content is taught in an understandable way. | 4 | 4 | 232 |
| The course is clearly structured in terms of content. | 4 | 4 | 232 |
| Learning objectives were clearly defined at the beginning. | 4 | 4 | 231 |
| The format of the courses with the elements used is well suited to achieve the learning objectives. | 4 | 4 | 231 |
| It was easy for me to follow the lectures and seminars held online. | 4 | 4 | 228 |
| Technical quality of teaching | | | |
| Teachers had no problems with technical implementation of online teaching formats. | 4 | 3 | 228 |
| Technical implementation of the course has worked well on my side (e. g. technical equipment, internet connection). | 5 | 4 | 228 |
| Technical implementation of the course has worked well on the part of faculty (e. g. setting / access for Moodle and Apps). | 4 | 4 | 230 |
| Organization of teaching | | | |
| The lectures of this course are well organized. | 4 | 3 | 229 |
| Dates, links and information about the courses and online materials were well communicated. | 4 | 4 | 233 |
| The ratio of synchronous (live) formats (video conferences with small group sharing) and asynchronous formats (lecture recordings, scripts, online assignments) was good. | 4 | 4 | 225 |
| Advantages of web-based learning | | | |
| The digital curriculum made me feel like I had a better overview of the content of my studies. | 3 | 3 | 230 |
| Advantages of web-based learning led to more efficiency in my learning. | 3 | 3 | 230 |
| Advantages of web-based learning gave me more flexibility in my life. | 5 | 5 | 230 |
| Due to different offers (seminars, lectures, screencasts…) I had the possibility to easily choose my individual learning preferences. | 5 | 4 | 229 |
| Subjective learning success | | | |
| My learning growth was high. | 4 | 4 | 230 |
| Without the personal contact to patients, I lacked the practical application of the newly learned. *(Negatively poled item)* | 5 | 5 | 228 |
| With the materials provided, I felt I was adequately prepared for the exam this semester. | 4 | 4 | 229 |
| With the materials provided, I feel I am adequately prepared for the state exam. | 2 | 3 | 226 |
| Interactions with students and teachers | | | |
| It was easy for me to ask organizational questions and get answers. | 3 | 4 | 221 |
| It was easy for me to ask content-related questions and get answers. | 3 | 4 | 216 |
| There are enough opportunities for exchange with other students within the framework of the courses. | 2 | 2 | 228 |
| There are enough opportunities for exchange with teachers during the courses. | 3 | 3 | 226 |
| Fears and changes due to pandemic situation | | | |
| Self-study has changed my daily rhythm. | 5 | 4 | 229 |
| This semester I have felt stress especially often in organizing the learning content. | 2 | 3 | 228 |
| Before the present exam I was burdened with more nervousness than in past exams. | 2 | 2 | 228 |
| Due to some last-minute changes in the curriculum to current COVID-19 management, I have noticed an uncertainty about my own studies. | 1 | 3 | 229 |
| Due to the publicly communicated possibility that the summer semester 2020 could have been cancelled, I was confronted with financial worries. | 1 | 1 | 227 |

*Annotations.* 1 = “do not agree”, 2 = “tend not to agree”, 3 = “partly agree”, 4 = “tend to agree”, 5 = “fully agree”
